# Altered Expression Levels of Angiogenic Peptides in the Carotid Body of Spontaneously Hypertensive Rats

**DOI:** 10.3390/ijms26041620

**Published:** 2025-02-14

**Authors:** Dimitrinka Y. Atanasova, Pavel I. Rashev, Milena S. Mourdjeva, Despina V. Pupaki, Anita Hristova, Angel D. Dandov, Nikolai E. Lazarov

**Affiliations:** 1 Institute of Neurobiology, Bulgarian Academy of Sciences, 1113 Sofia, Bulgaria; pavel_rashev@abv.bg; 2 Department of Anatomy, Faculty of Medicine, Trakia University, 6003 Stara Zagora, Bulgaria; 3 Institute of Biology and Immunology of Reproduction “Acad. Kiril Bratanov”, Bulgarian Academy of Sciences, 1113 Sofia, Bulgaria; mourdjeva@ibir.bas.bg (M.S.M.); dpupaki@ibir.bas.bg (D.V.P.); 4 Faculty of Medicine, Trakia University, 6003 Stara Zagora, Bulgaria; anita.hristova.22@trakia-uni.bg; 5 Department of Anatomy and Histology, Medical University of Sofia, 1431 Sofia, Bulgaria; adandov@medfac.mu-sofia.bg

**Keywords:** carotid body, endothelin-1, endothelin-converting enzyme, endothelin receptors, hypertension, spontaneously hypertensive rats, vascular endothelial growth factor, VEGF receptors, Wistar rats

## Abstract

The carotid body (CB), the main peripheral arterial chemoreceptor, exhibits considerable structural and neurochemical plasticity in response to pathological conditions such as high blood pressure. Previous studies have shown that morphological alterations in the hypertensive CB are characterized by enlarged parenchyma due to cellular hypertrophy and hyperplasia, and vasodilation. To test whether hypertension can also induce neoangiogenesis and modulate its chemosensory function, we examined the immunohistochemical expression of two angiogenic factors, vascular endothelial growth factor (VEGF) and endothelin-1 (ET), and their corresponding receptors in the CB of adult spontaneously hypertensive rats (SHRs), and compared their expression patterns to that of age-matched normotensive Wistar rats (NWR). We found an increased VEGF-A and B, and VEGFR-2 expression in glomus and endothelial cells in the enlarged CB glomeruli of SHRs compared with that in NWR. Conversely, weaker immunoreactivity to VEGFR-1 was detected in cell clusters of the hypertensive CB. The expression of endothelin-converting enzyme 1 and its receptor ETA was higher in a subset of glomus cells in the normotensive CB, while the immunoreactivity to the ETB receptor was enhanced in endothelial cells of CB blood vessels in SHRs. The elevated endothelial expression of VEGF and ET-1 suggests their role as local vascular remodeling factors in the adaptation to hypertension, though their involvement in the cellular rearrangement and modulation of chemosensory function could also be implied.

## 1. Introduction

The carotid body (CB), the main peripheral arterial chemoreceptor in humans, is a tiny anatomical structure with remarkable complexity that integrates information from various sources, processes it, and triggers adequate physiological responses. The CB registers the levels of gases in blood and responds to their changes by regulating breathing. It is strategically positioned in the vicinity of the common carotid artery bifurcation for monitoring blood chemicals before they reach the brain, an organ that is particularly sensitive to oxygen deprivation [1]. Its basic morphofunctional unit, known as glomerulus or glomoid, is a ball of highly vascular tissue, composed of two juxtaposed cell types: neuron-like type I or glomus cells and glial-like type II, also called sustentacular cells [2,3]. Glomus cells are considered the chemosensory cells of the organ that contain secretory granules packed with putative neurotransmitters and selectively express tyrosine hydroxylase (TH) [4]. Classically, the sustentacular cells are supporting cells which play a role in metabolic support, but they have recently been assumed to be stem cells that behave as glomus cell precursors [5,6].

One of the most striking morphological traits of the CB is its rich vascularization. Previous stereological studies have demonstrated that nearly one-fourth of the total CB volume in the cat is occupied by blood vessels and our microangioCT data have further shown that it is also true for the rat CB [3,7]. Moreover, it has been revealed that the CB as a single entity is one of the most irrigated organs per unit weight of any tissue in the body and its vascularization is 5–6-fold higher than that of the brain [4,8]. Fine blood vessels pierce the glomeruli, and a dense vascular bed of fenestrated capillaries forms around them. In contrast, a profuse capillary network travels in the walls of the connective tissue, which converges on the surface to create a fibrous organ capsule [9,10].

Another distinct structural feature of the CB is its dense innervation. It has been found that this highly vascular complex is dually innervated by sensory nerve fibers and autonomic nerve fibers of both sympathetic and parasympathetic origins. The afferent innervation of glomus cells is mainly provided by petrosal ganglion neurons of the glossopharyngeal nerve through the carotid sinus nerve, and it also comes from the superior (jugular) ganglion and inferior (nodose) ganglion cells of the vagus nerve [11]. The sympathetic nerve supply is relayed by postganglionic neurons from the closely located superior cervical ganglion via the ganglioglomerular nerve [12], while the parasympathetic innervation is received from vasomotor fibers of ganglion cells located around or even inside the CB, so the latter are collectively referred to as the (internal) carotid ganglion [11,13]. Most sympathetic nerve fibers are thought to supply blood vessels [14] and a few of them may also innervate glomus cells [11,15] while parasympathetic fibers are supposed to innervate smooth muscle cells of CB arteries or arterioles, and may also affect the glomus cell transmitter content and release [16,17]. It is believed that the presence of such an inhibitory efferent innervation, mediated by the release of nitric oxide, provides additional control of the afferent discharge [18].

In recent decades, CB dysfunction has been implicated in various physiological and pathophysiological conditions and its structure and neurochemical profile is altered in certain human sympathetic-related diseases, including essential hypertension. Hypertension is characterized by hypersensitivity to hypoxia and is associated with endothelial dysfunction, increased oxidative stress in blood vessels and activation of the inflammatory mediators [19,20]. Prior studies have indicated that the CB parenchyma is slightly enlarged in size in hypertensive rats than in age-matched normotensive controls, and that this enlargement is primarily due to glomus cell hypertrophy and hyperplasia, insignificant vasodilation and vascular remodeling but no neovascularization [21,22]. CB enlargement has also been observed post-mortem in patients with essential hypertension [23] and radiologically confirmed by computed tomographic angiography [24] and carotid ultrasonography [25].

In addition to the significant cellular rearrangement that culminates in structural plasticity, hypertension also induces neurochemical changes in chemosensory cells of the CB. Indeed, we have previously shown that the nitrergic metabolism of its cell population is impaired under hypertensive conditions, which may activate CB chemosensitivity [26]. Our research has further demonstrated that the components of the neurotrophin signaling system (growth factors and corresponding receptors) display an abnormally enhanced expression in the hypertensive CB which possibly modulates the chemosensory processing [21]. In addition, experimental evidence has suggested that the morpho-chemical changes in the rat CB exposed to chronic hypoxia may be associated with an increased production of angiogenic factors such as vascular endothelial growth factor (VEGF) and endothelin (ET-1) in both endothelial and chemoreceptor cells (reviewed in [27,28]).

Vascular endothelial growth factor (VEGF) is a key regulator of angiogenesis, vascular permeability, and endothelial cell survival, acting primarily through its receptor VEGFR-2 and to a lesser extent through VEGFR-1 and VEGFR-3 [29,30]. It plays a critical role in both physiological processes, such as embryonic development and tissue repair, and in pathological states, notably in cancer angiogenesis where tumor growth depends on neovascularization [31,32]. Endothelin-1 (ET-1) exerts its effects through endothelin type A (ETA) and endothelin type B (ETB) receptors, influencing the vascular tone, blood pressure regulation, and tissue remodeling [33,34]. ETA commonly promotes vasoconstriction and cell proliferation, whereas ETB can mediate vasodilation, yet it elicits vasoconstriction in smooth muscle cells [34]. Substantial crosstalk exists between the VEGF and ET-1 pathways; VEGF can stimulate the production of ET-1, and in turn ET-1 can reciprocally modulate angiogenesis by altering the VEGF expression or activity [35]. These interactions are especially relevant in tumor microenvironments, where both angiogenesis and vascular tone are dysregulated, and in the pathophysiology of cardiovascular diseases.

VEGF is primarily induced by hypoxia via hypoxia-inducible factor-1α (HIF-1α) and cytokines and is crucial for angiogenesis and vascular permeability [32]. ET-1, a potent vasoconstrictor, is upregulated by stress, angiotensin II, and various inflammatory mediators, significantly affecting the vascular tone and remodeling. Inflammation, through cytokines and chemokines, can increase or modulate both VEGF and ET-1 signaling [30,32]. Persistent inflammation disrupts normal vascular processes, leading to extensive remodeling marked by endothelial proliferation, smooth muscle migration, and adventitial changes.

Since chronic hypoxia is also known to be associated with elevated sympathetic activity and hypertension, and may cause angiogenesis in the CB, we tested the hypothesis that hypertension induces the upregulation of these two angiogenic peptides. This can mediate microvascular remodeling and affect the chemosensory function of the hypertensive CB. We studied the immunohistochemical expression of VEGF and ET-1 and their corresponding receptors in the CB of adult spontaneously hypertensive rats (SHRs), a well-known experimental model of hypertension. Then, we compared their expression patterns to that of age-matched normotensive Wistar rats (NWR).

## 2. Results

### 2.1. Vascular Endothelial Growth Factor

We examined the most important members of VEGF family, VEGF variant A (VEGF-A) and B (VEGF-B) which promote angiogenesis and vascular permeability by activating two receptors, VEGFR-1 and VEGFR-2.

**Figure 1:**
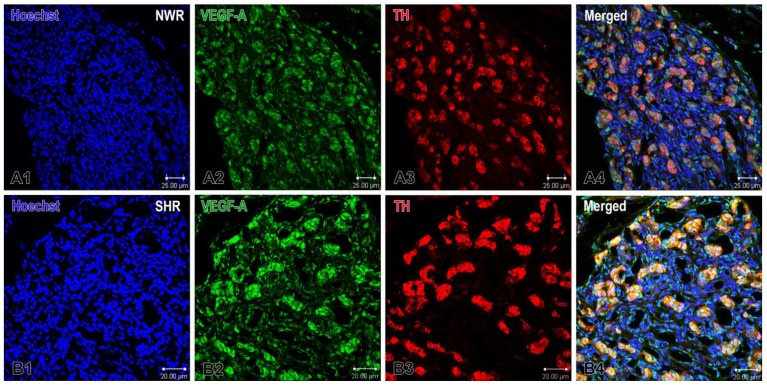
Immunofluorescence images demonstrating VEGF-A expression in the carotid body of normotensive Wistar rats (NWR) and spontaneously hypertensive rats (SHR). Panels (**A1**–**A4**) show VEGF-immunostained glomus and endothelial cells (green), TH-immunoreactive glomus cells (red), and co-localization of VEGF-A and TH positive cells in confocal dual-color images (orange). The cell nuclei are counterstained with Hoechst (blue). Panels (**B1**–**B4**) show images of the hypertensive CB. Note the enhanced VEGF-A expression in glomus cells in the merged image (**B4**). Original magnification ×40, scale bars, 25 µm (**A1**–**A4**), 20 µm (**B1**–**B4**).

Immunofluorescence and confocal microscopy revealed a strongly increased protein expression of both VEGF-A and VEGF-B in glomus and endothelial cells in the enlarged CB glomeruli of SHRs compared with that in NWR. Specifically, we observed that a subset of glomus cells and some endothelial cells in the CB were immunoreactive to VEGF-A and immunostaining of the glomus cells was stronger in the hypertensive than that in normotensive rats (Figure 1). We calculated the correlation index between TH and VEGF-A in both animal strains using the ImageJ colocalization plugin. In normotensive animals, the correlation index was 0.47, with the mean threshold value for TH being 34.23 and the mean threshold value for VEGF-A being 34.51. In hypertensive animals, the correlation index was 0.60, the mean threshold values for TH were 48.28, and for VEGF-A were 54.11. These results imply that hypertensive animals show both a higher expression (as inferred by higher threshold values) and a higher colocalization (correlation index) of TH and VEGF-A, pointing to a potentially significant interaction between sympathetic innervation and angiogenic pathways in the context of hypertension.

**Figure 2:**
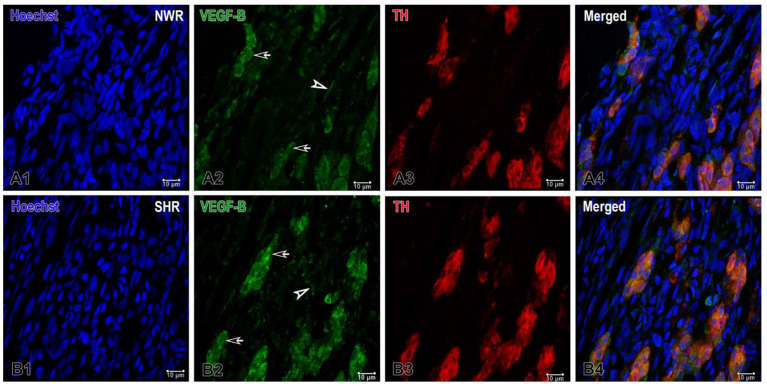
Representative images of double immunofluorescence staining for VEGF-B (green), TH (red) and Hoechst (blue as a nuclear counterstain) in the normotensive and hypertensive carotid body. (**A1**–**A4**) Immunostained sections of the carotid body depicting the expression of VEGF-B in glomus (arrows) and endothelial (arrowhead) cells in normotensive Wistar rats (NWR). A few endothelial cells are immunostained for this protein (**A2**) and a subset of co-stained glomus cells (orange) are visualized in a merged image (**A4**). (**B1**–**B4**) Representative images of the carotid body in spontaneously hypertensive rats (SHR) showing an increased expression of VEGF-B in glomus cells (**B4**). Original magnification ×63, scale bars equal 10 µm.

Similarly, VEGF-B was expressed in a few endothelial and several glomus cells in the CB of NWR. The visual examination of these sections revealed that its expression was higher in glomus cells of the enlarged glomeruli in the hypertensive CB compared with that in the normotensive CB, as highlighted by double-immunostaining with TH (Figure 2). In normotensive animals, the correlation index between TH and VEGF-B was 0.49, with mean threshold values of 29.05 for TH and 18.43 for VEGF-B. In hypertensive animals, the correlation index was slightly higher at 0.54, with mean thresholds of 32.12 for TH and 19.67 for VEGF-B. These values indicate a moderate degree of colocalization between TH and VEGF-B in both groups, with a marginally higher correlation observed in the hypertensive group compared to the normotensive group.

**Figure 3:**
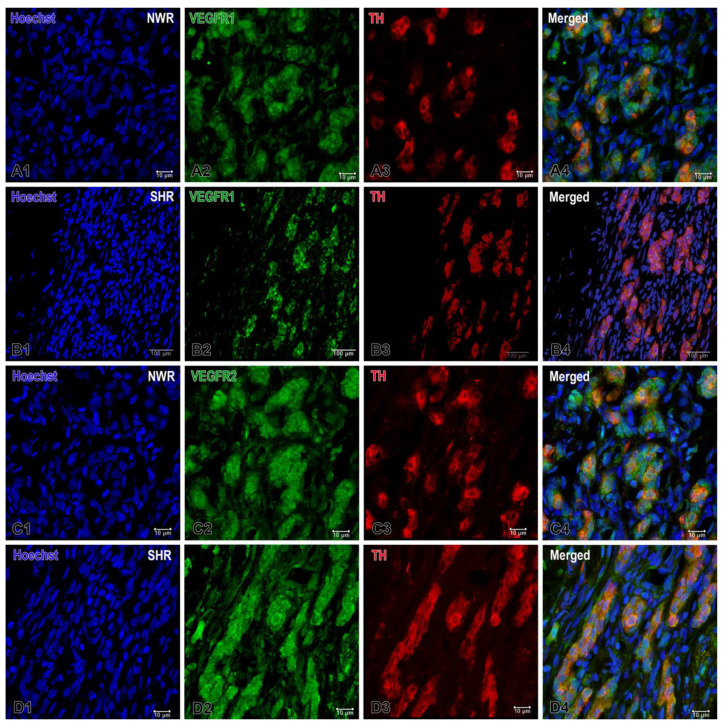
Representative images of immunofluorescence staining for VEGFR1 (**A**,**B**) and VEGFR2 (**C**,**D**) in the normotensive (**A1**–**A4**;**C1**–**C4**) and hypertensive carotid body (**B1**–**B4**;**D1**–**D4**). Double immunofluorescence staining analyzed by confocal laser scanning microscopy demonstrates VEGFR1, VEGFR2 (green), TH (red) and co-localization (orange) of VEGFR1-VEGFR2/TH in overlapping images. The cell nuclei are counterstained with Hoechst (blue). Note the highly increased expression for VEGFR1 in the glomus cells of NWR (**A4**) compared to that in SHR (**B4**). A slightly increased immunostaining for VEGFR2 is also observed in some glomus cells in SHR (**D4**) compared to the immunostained glomus cells in NWR (**C4**). Original magnification ×40 for (**B**) and ×63 for (**A**,**C**,**D**), scale bars, 10 µm (**A**,**C**,**D**), 100 µm (**B**).

Accordingly, a markedly increased expression of the receptor VEGFR1 was observed in the endothelial cells and in some glomus cells in the CB glomeruli of NWRs, while a relatively weaker but still enhanced expression of VEGFR2 was detected in cell clusters of the hypertensive CB compared to that in normotensive rats (Figure 3). In addition to the apparent expression of VEGFR1 on endothelial cells, a higher expression of this receptor was also seen in glomus cells of the normotensive CB. On the other hand, a slightly higher and statistically significant expression of VEGFR2 was found in glomus cells of SHRs compared to that in the normotensive group.

Our ImageJ colocalization data indicated a moderate overlap (0.53) of TH and VEGFR1 in normotensive animals, which significantly decreased (0.19) under hypertensive conditions. The threshold values suggest an increase in the intensity of TH in hypertensive tissues, while VEGFR1 thresholds remain relatively stable. These findings are consistent with the idea that hypertension involves altered neurovascular or sympathetic regulation pathways, reflected by the reduced spatial coupling of these two markers. A correlation index of 0.56 in normotensive animals indicated moderate colocalization of TH and VEGFR2. A correlation index of 1 in hypertensive animals suggests a nearly perfect overlap, implying that wherever TH is present, VEGFR2 is also present in a very similar intensity range. The matching and also higher threshold values in the hypertensive group hinted at a stronger and possibly upregulated, and more synchronous expression of these two markers under hypertensive conditions.

**Figure 4:**
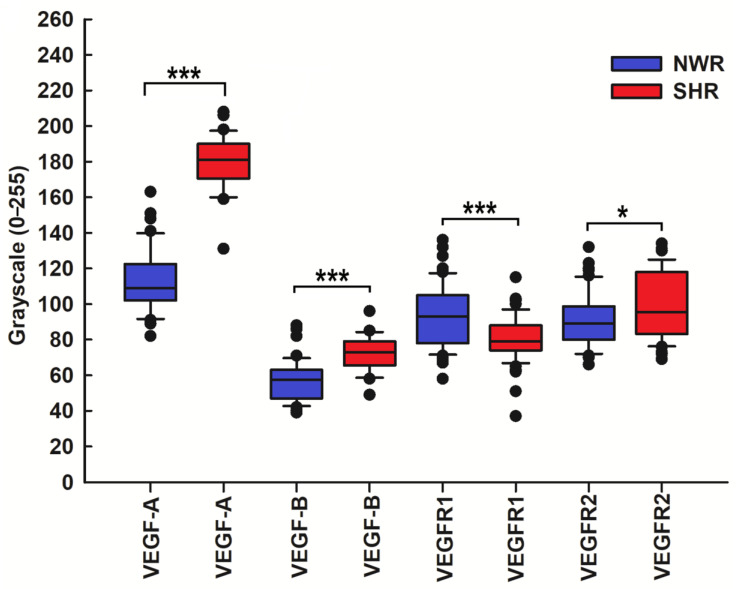
Statistical comparison of the immunostaining intensity for VEGF-A, VEGF-B, and VEGF receptors in NWRs (blue boxes) and SHRs (red boxes). Note that the immunostaining intensity for the VEGF-A in the chemosensory glomus cells of the SHR was stronger (median, 181 versus 109, *** *p* ≤ 0.001) than that observed in NWR rats. An enhanced and statistically significantly increased expression of vascular endothelial growth factor-B (VEGF-B) (median, 73 versus 57.5, *** *p* ≤ 0.001) and its receptor VEGFR2 (median, 95.5 versus 89, * *p* = 0.036) in glomus cells of SHR compared with NWR controls was also observed, as highlighted by the double labeling technique with their marker tyrosine hydroxylase (TH). Vascular endothelial growth factor receptor 1 (VEGFR1) is much less expressed in glomus cells of hypertensive CB compared to age-matched NWR controls (median, 79 versus 88.5, *** *p* ≤ 0.001).

The statistical analysis revealed an altered and significantly increased expression of VEGF-A and VEGF-B and their receptor VEGFR2 in glomus cells of CB glomeruli in SHRs, compared with NWR controls, as highlighted by the double immunofluorescence technique with the glomus cell marker TH. Conversely, VEGFR1 was much less expressed in glomus cells of hypertensive CB compared to age-matched controls (Figure 4).

### 2.2. Endothelin

Endothelin-1 (ET-1) is another vasoactive peptide, and it is one of a family of three endothelins that exert their action through two G-protein-coupled transmembrane receptors, ETA and ETB.

**Figure 5:**
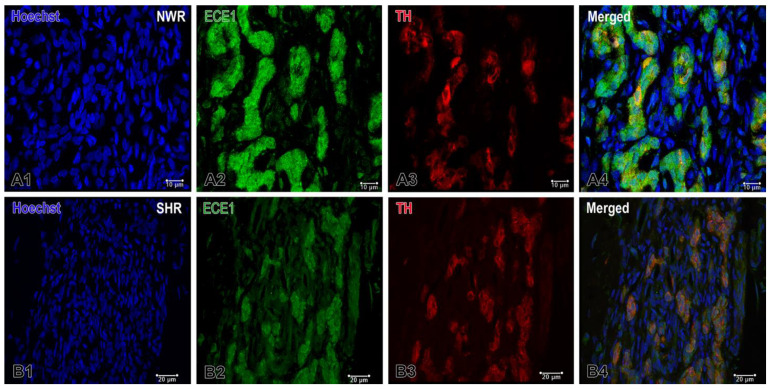
Double immunofluorescence staining of endothelin-converting enzyme 1 (ECE1) in the normotensive (**A**) and hypertensive (**B**) carotid body. (**A1**–**A4**) Confocal microscope images showing the high enzymatic activity in the endothelial cells and in a few glomus cells in the carotid body of NWR. TH and ECE1 colocalization in glomus cells can be seen in an overlaying image (**A4**). (**B1**–**B4**) ECE1 is weakly expressed only in some glomus cells in the carotid body of SHR. Original magnifications ×63 for (**A1**–**A4**) and ×40 for (**B1**–**B4**), scale bars, 10 µm (**A**), 20 µm (**B**).

Immunofluorescence staining detected the activity of endothelin-converting enzyme 1 (ECE1), which was high in the endothelial cells and in some glomus cells in the normotensive CB, while it was either undetectable or expressed at low levels in the latter under hypertensive conditions (Figure 5). The correlation index rose from 0.53 to 0.74 in hypertensive animals, indicating a stronger overlap (colocalization) of TH and ECE1 signals compared to normotensive controls. This suggests that in hypertensive conditions, TH and ECE1 are more likely to be expressed or localized in the same regions or cellular compartments. In normotensive animals, the plugin set much higher threshold levels for ECE1 (44.38) compared to TH (16.82). In hypertensive animals, both TH (13.87) and ECE1 (14.59) thresholds were relatively close and markedly lower than their normotensive counterparts, especially for ECE1 (from 44.38 down to 14.59). An elevated correlation index in hypertensive animals might point to a pathological interplay between TH, a key enzyme in catecholamine synthesis, and ECE1 that is involved in the endothelin production. This could suggest that the hypertensive state involves a closer functional or spatial interaction between the catecholaminergic pathway and the endothelin system.

**Figure 6:**
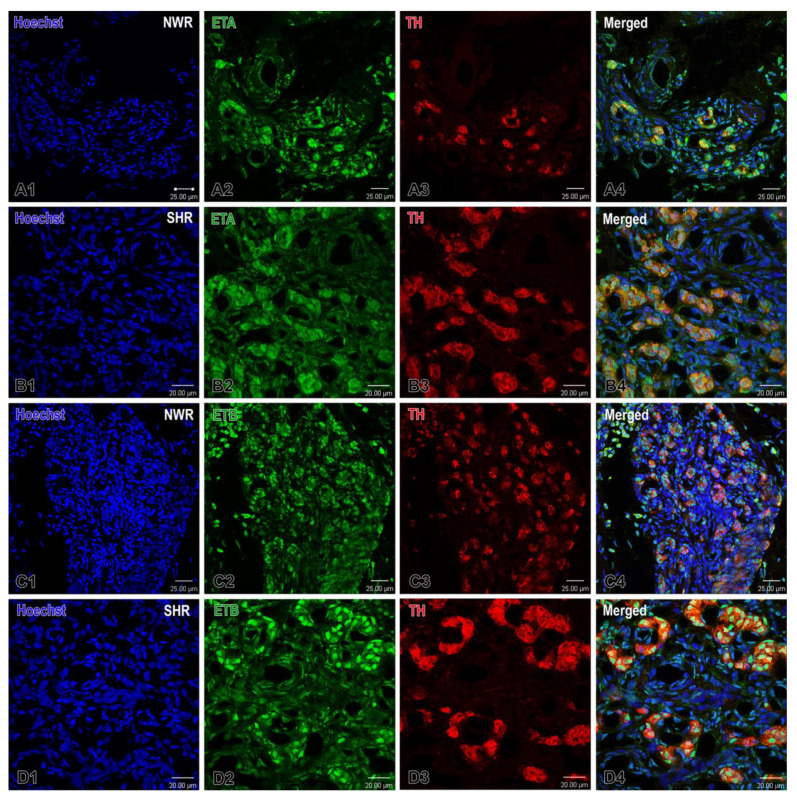
Confocal images illustrating the expression of endothelin receptors ETA and B (green) in the carotid body of normotensive Wistar rats (NWR) (**A1**–**A4**;**C1**–**C4**) and spontaneously hypertensive rats (SHR) (**B1**–**B4**;**D1**–**D4**). (**A1**–**A4**) Note almost unchanged expression for ETA (orange) in the glomus cells of SHR in a merged image (**B4**) compared to that in NWR (**A4**). The cell nuclei are counterstained with Hoechst (blue). Immunofluorescence staining for TH (red) is used to detect glomus cells. An increased immunostaining for ETB is observed in some glomus cells in SHR (**D4**) than in the immunopositive glomus cells in NWR (**C4**). Original magnification ×40 for (**A**,**C**) and ×63 for (**B**,**D**), scale bars, equal 25 µm (**A**,**C**), 20 µm (**B**,**D**).

The double immunofluorescence procedure further identified that the immunostaining for its receptor ETA in glomus cells was not significantly different between the two breeds, while the immunoreactivity to the ETB receptor was somewhat higher, predominantly in endothelial cells of CB perilobular blood vessels in hypertensive rats (Figure 6). The slightly lower correlation index in hypertensive animals (0.61 vs. 0.64) indicated an insignificant reduction in the degree of TH–ETA colocalization or co-expression. However, this difference was relatively modest, suggesting that TH and ETA remain moderately correlated in both groups. Interestingly enough, the calculation of the correlation index between TH and ETB shows a notable consistency of 0.49 in both normotensive and hypertensive animals. Although the overall correlation between TH and ETB remained the same (0.49), the threshold values in the hypertensive group were higher than in the normotensive group. In ImageJ-based colocalization analysis, these thresholds often reflected the intensity levels above which pixels were considered part of the “positive” signal for each marker. Therefore, the higher thresholds in hypertensive animals may indicate an increased expression or signal intensity of TH and/or ETB in hypertensive animals, leading to higher overall fluorescence or staining that shifts the threshold upward.

**Figure 7:**
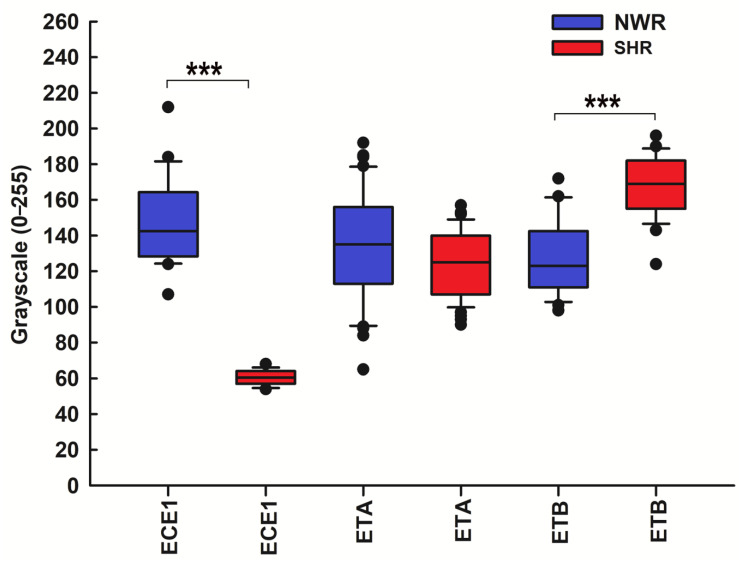
Statistical comparison of the immunostaining intensity for ECE1 and ET receptors in NWR (blue boxes) and SHR (red boxes). Note that when compared with the control CB, the staining intensity for ECE1 was lower in the immunoreactive cells in the CB of SHR (median, 60.5 versus 142.5, *** *p* < 0.001). We were unable to detect statistically significant differences in ETA expression in hypertensive versus normotensive CBs (median, 125 versus 135, *p* = 0.068). Immunoreactivity for the endothelin receptor type B (ETB) was localized in glomus cells in both the normotensive and hypertensive CB. Compared to the control CB, the staining intensity for ETB was higher in the immunoreactive cells in the CB of SHR (median, 169 versus 123, *** *p* < 0.001).

The statistical analysis of ECE1 and ET receptor staining intensity yielded similar results. Specifically, no statistically significant differences in ETA expression in hypertensive versus normotensive CBs were detected, whereas the staining intensity for ECE1 was significantly higher in the immunoreactive cells in the CB of NWR compared to that in SHRs. On the contrary, the intensity of immunostaining for ETB was considerably higher in the immunoreactive cells in the hypertensive CB than in the normotensive control CB (Figure 7).

## 3. Discussion

Consistent with its ability to sense blood-borne chemicals, the CB is a highly vascularized organ, and its intraorgan hemodynamics possibly play a role in the process of chemoreception. The dysregulation of the CB function and altered oxygen saturation are implicated in the pathophysiology of various human cardiovascular diseases, including hypertension (reviewed in [36,37]). Certainly, it has been suggested that the CB input plays a fundamental role in both the genesis and maintenance of hypertension [38]. Much of the available evidence has further demonstrated that hypertension induces marked morphological and neurochemical changes within the CB in response to the elevated systemic blood pressure and concomitant hypoxia (for recent reviews, see [22,39,40,41]). Specifically, it has been shown that the volume of the CB parenchyma and its vasculature are influenced by hypertension in both patients with primary hypertension [42] and SHR [39]. Such structural alterations in the CB are observed during the developmental stages of the disease and could be considered adaptive changes. However, they are probably caused by another factor rather than hypertension per se because it is unlikely that blood pressure would remain high in a structure whose microvasculature is mostly composed of fenestrated capillaries closely associated with cell clusters [43]. Indeed, it has recently been suggested that these changes in the SHR strain could be ascribed to the increased sympathetic vasomotor tone under hypertensive conditions [44]. Moreover, increased sensitivity of the arterial chemoreceptor drive has also been reported in human hypertensive subjects [45] and SHR [46], and this is reviewed in [47].

The present results show that the two examined vasoactive peptides, VEGF and ET-1, are largely expressed in the CB of both normotensive and hypertensive rats. The expression of VEGF protein and its receptors demonstrate almost identical staining patterns. This is because both VEGF-A and VEGF-B are localized in endothelial cells and a few glomus cells in the CB glomeruli of normotensive controls and their expression levels, including VEGFR1 expression, are significantly enhanced in the hypertensive CB. Previous studies have immunohistochemically shown an increased expression of VEGF and VEGFR1 by glomus cells in the CB of chronically hypoxic rats [48,49,50]. Similar upregulation of VEGF has also been reported in cell clusters in a rat model of L-NAME-induced hypertension [51]. It seems that VEGF is markedly distributed throughout glomus cells and blood vessels in the CB, and its expression is evidently increased under hypertensive conditions. It is likely that VEGF specifically binds to VEGFR1 on both the glomus and endothelial cells to regulate hyperplasia of the glomus cells and induce endothelial cell proliferation, thus promoting angiogenesis and neovascularization in the hypertensive CB.

ET-1 is a potent vasoconstrictor peptide with a direct angiogenic effect on endothelial and perivascular cells which may also enhance the chemosensory discharge in the CB. ET-1 immunoreactivity has been observed in blood vessels and glomus cells of the normotensive rat CB [52] and, moreover, its expression there has been reported to be considerably upregulated by chronic hypoxia [53,54]. Chen et al. (2002) have further shown that ET-1 may enhance chemosensory activity to hypoxia [53,54], while others have ascribed this effect to local vasoconstriction, though these authors do not exclude paracrine action of the peptide on glomus cells [55,56]. Upregulation of the ETA receptor after exposure to chronic hypoxia has been reported as well [52,53,54], while chronic intermittent hypoxia causes an increase in the ETB receptor in glomus cells [56,57]. Our results provide immunohistochemical evidence that hypertension may also induce an upregulation of ET-1 expression by glomus cells and its excitatory effects on chemoreception are mediated by ETB receptors.

Such an upregulated expression of VEGF and ET-1 suggests a paracrine role of these angiogenic peptides in the modulation of CB function and plasticity which could potentially play a pivotal role in cellular adaptation to hypertensive state. As recently suggested, CB hyperexcitability is driven by its own sympathetic innervation [58], and thus the heightened chemosensory discharge causes sympathetic neural activation which in turn could contribute to the development of hypertension [59]. On the other hand, it has been established that hypertension is abolished by CB removal [38], and that the bilateral CB ablation in some hypertensive patients causes an immediate and sustained fall in the blood pressure [60] and lowers it in SHRs [61], thus suggesting that selective carotid glomectomy may be an effective procedure for the treatment of resistant hypertension [60,62].

## 4. Materials and Methods

### 4.1. Experimental Animals

The experiments were carried out on adult (12-week-old; n = 8) SHRs and age-matched normotensive Wistar rats (NWR; n = 8) of both sexes (equal numbers n = 4 for males and females) weighing 220–260 g. The experimental animals were bred and provided by the vivarium of our institute, where the blood pressure of both SHR and NWR was measured noninvasively by the tail cuff method and the value for each rat was the mean of three measurements. In the SHRs, the systolic blood pressure was above 150 mmHg and the diastolic more than 90 mmHg, so they met the reference value to classify them as hypertensive, while the blood pressure values of the NWR were less than 120/80 mmHg, respectively. All experimental procedures adhered to the ethical guidelines of the EU Directive 2010/63/EU for the protection of animals used for scientific purposes following a standard protocol established by the Bioethical Commission of the Biomedical Research at the Institute of Neurobiology of the Bulgarian Academy of Sciences, and was approved by the Bulgarian Food Safety Agency (Approval Protocol No. 295 of 13 April 2021). All efforts were made to minimize the number of animals used and their suffering.

### 4.2. Tissue Preparation

The rats were deeply anesthetized with an intraperitoneal injection of sodium thiopental (30 mg/kg, Sigma-Aldrich, Chemie GmbH, Taufkirchen, Germany) and transcardially perfused first with 0.05 M phosphate-buffered saline (PBS), followed by 4% paraformaldehyde (CAS: 30525-89-4, Sigma-Aldrich, Vienna, Austria) in 0.1 M phosphate buffer (PB), pH 7.36. After perfusion, the carotid bifurcations were quickly removed, both CBs were immediately dissected out and tissue samples were postfixed in the same fixative overnight at 4 °C. Thereafter, the tissue blocks were washed in tap water, dehydrated, embedded in paraffin, and cut into 6 μm thick sections using a paraffin microtome (Leica RM 2155, Wetzlar, Germany). The first section of each series was stained with hematoxylin and eosin to inspect CB morphology, and the remaining sections were used for immunofluorescence.

### 4.3. Immunofluorescence

All samples from both SHR and NWR were passed through the immunofluorescence procedure simultaneously to avoid variations in the immunostaining handling process. Tissue sections from both normotensive and spontaneously hypertensive rats were processed at the same time under the same experimental conditions. Initially they were deparaffinized with xylene and rehydrated in a descending ethanol series, 100%, 95%, 90%, 80%, 70% alcohol and distilled water for 5 min each. After an antigen retrieval in water bath thermostat WB-4MS at a temperature of 95 degrees for 20 min in 0.01 M citrate buffer, pH 6.0, the tissue sections were cooled and washed three times in Tris buffered saline with 0.05% Tween-20 (TTBT), pH 7.6 for 5 min each. Then, they were incubated in Super Block (ScyTek Laboratories Inc., Logan, UT, USA) for 5 min at room temperature. After washing three times in TTBS for 5 min each, the sections were incubated with the primary antibodies overnight at 4 °C. Table 1 shows the primary and secondary antibodies used, the appropriate working dilutions and the supplier companies.

After the incubation, the tissue slides were washed in TTBS and incubated with the secondary antibodies: Goat Anti-Mouse IgG (H + L) (Elab Fluor® 594 conjugated) (E-AB-1059, Wuhan Elabscience Biotechnology Co., Ltd., Wuhan, China) and Goat Anti-Rabbit IgG (H + L) (Elab Fluor® 488 conjugated) (E-AB-1055, Wuhan Elabscience Biotechnology Co., Ltd., China) for 1 h at room temperature. Following three washes in TBS (E-IR-R116, Wuhan Elabscience Biotechnology Co., Ltd., China) for 5 min each, the sections were incubated with Hoechst 33342 (sc-391054, Santa Cruz Biotechnology Inc., Dallas, TX, USA) for 10 min, then rinsed three times in TBS for 5 min each. Finally, the sections were mounted with FluoreGuard Mounting Medium (Hard Set) (FMH030—ScyTek Laboratories Inc., Logan, UT, USA) and coverslipped. The slides were observed on a Leica TCS SPE confocal microscope (Wetzlar, Germany) equipped with a Leica Application Suite X (LAS X) Microscope Software, Version 3.5.7.23225 (Leica Microsystems GmbH).

### 4.4. Image Analysis and Statistics

**Figure 8:**
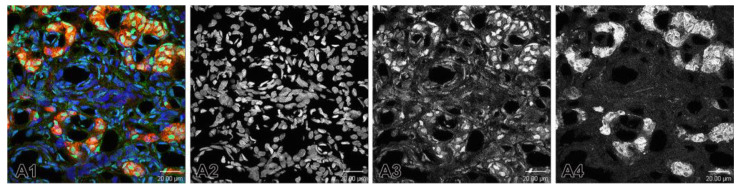
(**A1**) A merge image showing the nuclei of the cells that are counterstained with Hoechst (blue). Immunofluorescence staining for TH (red) is used to detect glomus cells. The immunostaining of ETB is observed in some glomus cells in SHR. (**A2**–**A4**) Example of the application of Channel Mixer Method and converting fluorescent color to grayscale. The monochrome box was checked before adjusting to blue (**A2**), green (**A3**), and red (**A4**) channels. Original magnification ×63, scale check bars, 20 µm.

**Figure 9:**
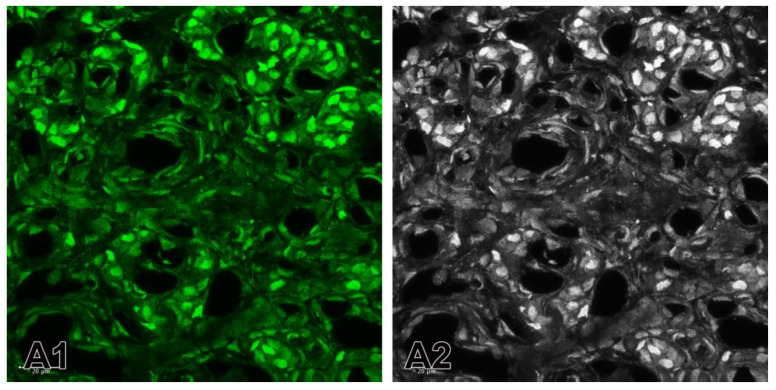
Example of the application of the split channels method. (**A1**) The split green channel for the expression of ETB in the carotid body of SHRs. (**A2**) Converted fluorescent color image to grayscale image. Original magnification ×63, scale bars 20 µm.

Specimens from both groups were then photographed under identical microscope settings. After the reactions, the stained and processed sections were digitalized using a Leica TCS SPE confocal microscope (Wetzlar, Germany) and the images were taken precisely on objective lenses 20×, 40× and 63×. Photomicrographs of the immunostained fluorescent tissue sections were converted into grayscale images. We applied two widely used methods for converting fluorescence microscopy images to grayscale: Channel Mixer Method (Figure 8) and Split Channels Method (Figure 9). In order to deal with the troubleshooting, in the first method we always checked the Monochrome box at the bottom left of the Channel Mixer dialog box before adjusting to red (Figure 8(A4)), green (Figure 8(A3)) and blue (Figure 8(A2)) channels.

In the second method, we used split channels (Figure 9). Bearing in mind that an image with layers cannot be split into channels, we made sure that the image did not have layers. Thus, if there were layers, we had to merge them and then split the channels.

The grayscale intensity (range 0–255: black = 0, white = 255) was defined and measured using the ImageJ Version 1.54 analysis program (National Institutes of Health, Bethesda, MD, USA). The proportion of immunopositive cells in the two experimental groups and their grayscale intensity were given as medians. The data from the statistical analysis and the comparison between the two breeds of animals were displayed as box-and-whisker plots. The central line in each box plot represented the median, whereas the lower and upper halves of the box were the 25th and 75th percentiles, respectively. The whiskers show each outlier for the group. The expression of substances was compared using a statistical program for data processing SigmaStat^®^ 11.0 software package (Systat Software, Inc., Berkshire, UK). The expression levels between the two breeds were evaluated by Student’s *t*-test for parametric data distribution or by Mann–Whitney U test to nonparametric data. Differences were considered statistically significant if *p*-values were <0.05.

## 5. Conclusions

In conclusion, the elevated expression of VEGF and ETB and the downregulation of VEGFR1, VEGFR2, and ECE1 in rat glomus cells of the hypertensive CB suggest a paracrine role of these angiogenic peptides in the modulation of its function and plasticity under hypertensive conditions. Obviously, the altered transmitter phenotype of CB chemoreceptor cells and the upregulated production of angiogenic factors may modulate the chemosensory processing in hypertensive animals which elicits sympathetic hyperactivity, consequently leading to elevated blood pressure. Moreover, since hypertension is known to increase CB vasoconstriction and vascular permeability, their involvement as local vascular remodeling factors in the adaptation to hypertension could also be implied.

## Figures and Tables

**Table 1:** List of primary and secondary antibodies used for immunofluorescence.

Name of Antibody	Company (Catalog No.)	Mono/ Polyclonal	Source	Dilution for IF
Vascular endothelial growth factor A (C-1) (VEGF-A)	Santa Cruz Biotechnology	Monoclonal	Mouse	1:50
Vascular endothelial growth factor B (VEGF-B)	Elabscience^®^ (E-AB-65950)	Polyclonal	Rabbit	1:400
Vascular endothelial growth factor receptor 1 (VEGFR1)	Elabscience^®^ (E-AB-65963)	Polyclonal	Rabbit	1:1000
Vascular endothelial growth factor receptor 2 (VEGFR2)	Elabscience^®^ (E-AB-63481)	Polyclonal	Rabbit	1:800
Tyrosine hydroxylase (TH)	Elabscience^®^ (E-AB-70206)	Monoclonal	Mouse	1:800
Endothelin converting enzyme 1 (ECE1)	Elabscience^®^ (E-AB-14052)	Polyclonal	Rabbit	1:400
Endothelin receptor type A (ETA)	Affinity Biosciences (DF4923) Cincinnati, OH, USA	Polyclonal	Rabbit	1:100
Endothelin receptor type B (ETB)	Affinity Biosciences (DF7104) Cincinnati, OH, USA	Polyclonal	Rabbit	1:300
Goat anti-mouse IgG (H + L) (Elab Fluor^®^ 594 conjugated)	Elabscience^®^, (E-AB-1059)	Polyclonal	Goat	1:100
Goat anti-rabbit IgG (H + L) (Elab Fluor^®^ 488 conjugated)	Elabscience^®^, (E-AB-1055)	Polyclonal	Goat	1:100

## Data Availability

All data generated or analyzed during this study are included in this article. Further inquiries can be directed to the corresponding author.
